# Microwave Ablation Compared With Laser Ablation for Treating Benign Thyroid Nodules in a Propensity-Score Matching Study

**DOI:** 10.3389/fendo.2019.00874

**Published:** 2019-12-13

**Authors:** Yi-Fan Shi, Ping Zhou, Yong-Feng Zhao, Wen-Gang Liu, Shuang-Ming Tian, Yong-Ping Liang

**Affiliations:** Department of Ultrasound, The Third Xiangya Hospital, Central South University, Changsha, China

**Keywords:** benign thyroid nodule, percutaneous thermal ablation, laser ablation, microwave ablation, ultrasound

## Abstract

**Objective:** The efficacy and safety of percutaneous ultrasound-guided microwave and laser ablation (MWA and LA, respectively), for treating benign thyroid nodules (BTNs), were retrospectively compared.

**Methods:** Patients (*n* = 318) underwent ablation of 328 BTNs. Confounding bias was reduced by propensity-score matching, and finally the MWA and LA groups each comprised 160 nodules. At baseline (before ablation), 3, 6, and 12 months, and every 6 months thereafter, the following were recorded: nodule volume reduction rate (VRR), neck symptom scores, cosmetic scores, complications, and side effects.

**Results:** The baseline characteristics of the MWA and LA groups were comparable. The volumes of all nodules were less at all follow-ups relative to the baseline, as were the symptom and cosmetic scores at postoperative 6 months and thereafter (*P* < 0.01). At each follow-up, the overall VRRs of the MWA and LA groups were comparable. However, for nodules ≥13 mL, the VRR associated with LA at ≥6 months was significantly greater than that of MWA. The average ablation time for MWA was less than that of LA (*P* < 0.01). The overall incidences of major complications, minor complications, and side effects were 1.6, 2.2, and 18.4%, respectively, and there were no significant differences between the MWA and LA groups.

**Conclusion:** Percutaneous ultrasound-guided MWA and LA are both safe and effective for the treatment of BTNs. Each can significantly reduce the nodule volume and improve the neck symptoms and appearance of patients, with a low incidence of adverse side effects. The efficiency of MWA is higher than that of LA. For nodules ≥13 mL, MWA may be preferred, but at 6 months and subsequent follow-ups the reduction in volume was greater in patients receiving LA.

## Introduction

Thyroid nodules (TNs) are commonly encountered in the clinical practice, and affect 20–76% of the general population as shown by ultrasound (1). Most of these TNs (85–95%) are benign (BTNs), confirmed by fine-needle aspiration biopsy (2). Although not malignant, some patients with BTNs may require surgical treatment for aesthetic reasons, subjective symptoms, or concerns for potential growth and changes (3, 4). Surgical cure rates are high, but complications can include long hospitalization, upper airway obstruction, recurrent laryngeal nerve palsy, iatrogenic hypothyroidism, scarring, and difficulty in reoperation (5). Alternatively, the efficacy of thyroid hormone suppression is limited for reducing an enlarged thyroid gland, and such therapy may lead to low serum levels of thyroid stimulating hormone (6).

For many years, minimally invasive treatments for patients with BTNs have included either ultrasound-guided percutaneous laser or microwave ablation (LA and MWA, respectively). Studies have confirmed that both LA and MWA have high rates of volume reduction (VRR), with good preservation of thyroid function and few complications. Yet, to the best of our knowledge, there has been no systematic study that compared the clinical efficacy and safety of MWA and LA.

To provide more informed guidance for clinical decision-making, the present study retrospectively compared the clinical outcomes of single-session MWA with LA for patients with BTNs.

## Methods

### Patient

The Ethics Committee of Third Xiangya Hospital of Central South University approved this retrospective study and waived the requirement of written informed consent. Each patient provided written informed consent before MWA or LA after full explanation of the purpose and nature of the procedure used. The ablations were performed in accordance with approved guidelines and regulations, in a dedicated interventional operating room by a doctor with 10 years' experience.

For this study, all the patients conformed to the following inclusion criteria: a nodule that was >50% solid and benign, confirmed by cytologic examination or histopathological biopsy; with neck symptoms, cosmetic problems, or refused surgery or clinical observations, requiring minimally invasive interventional therapy with absolute informed consent; and serum levels of thyrotropin and thyroid hormone within normal limits. Cytologic examination was conducted in accordance with the American Bethesda System for Reporting Thyroid Cytopathology. Patients with any of the following were excluded from this study: medication or other treatment for TNs after ablation; incomplete data, or follow-up shorter than 6 months.

### Equipment

The ultrasound system was a MyLab Twice color Doppler ultrasound system (Esaote, Italy) equipped with contrast-enhanced ultrasound imaging technology. High-resolution linear probes (6–12 MHz) were used to monitor and direct the cytological or histological examination, pre-ablation assessment, ablation therapy, and follow-up.

MWA was performed with a KY-2000 2450 MHz microwave system (KY-2000, Kangyou Medical, Nanjing, China) produced by Nanjing Kangyou Medical, equipped with a 16-gauge, Teflon-coated, internal-cooled microwave antenna with a 3-mm active tip and a 10-cm shaft. This equipment was specifically modified for the ablation of TNs.

LA was conducted with an ultrasonic laser integrated system produced by Italian Esaote Medical, and Echolaser X4 laser treatment system. The device comprised a 1,064-μm diode laser unit with a maximum of 4 laser sources, each with an individual energy emission setting and independent activation, 0.3-mm diameter optic fiber, 21-gauge Chiba needle, and a foot pedal.

### Pre-Ablation Evaluations

To determine the diagnosis of TNs before ablation, a cytologic examination by fine-needle aspiration biopsy was necessary. In the event of a non-diagnostic cytology result, an additional histopathological examination by core-needle biopsy was conducted (7, 8). The ultrasound examination was indispensable for identifying and classifying the target nodule and characterizing the anatomical relationship between the nodule and the important surrounding structures.

The maximum diameter and two orthogonal diameters, echoic characteristics, internal blood flow distribution, and the ratio of solid components of each nodule were obtained by an experienced sonographer. The nodule volume (V) was estimated by the ellipsoid formula V = π*abc*/6, where *a* is the maximum diameter, and *b* and *c* are the 2 orthogonal diameters. Prior to ablation, the nodules were classified as either large or small (nodular volume ≥13 or <13 mL, respectively), as in prior studies (9–11).

The neck symptom scores were self-assessed by patients with scores ranging from 0 (nil) to 10 (most severe). Neck symptoms included pain or discomfort, foreign body sensation, and compression. The cosmetic scores were evaluated by an experienced physician and ranged from 1 to 4 as follows: 1, no palpable mass; 2, palpable mass but no cosmetic problems; 3, cosmetic problem on swallowing only or detected by an experienced physician; and 4, a readily detected cosmetic problem.

Laboratory tests were conducted prior to ablation, consisting of a complete blood count and blood coagulation, and the following thyroid function indicators: thyroid stimulating hormone, triiodothyronine (T3), free thyroxine (FT4), and calcitonin.

### Ablation Procedure

To compare the clinical applications of MWA and LA objectively, the nodules which underwent a single treatment session were chosen, and the clinical results were followed, recorded, and analyzed. If ≥2 target nodules of a patient were located in the 2 lobes of the thyroid, 2 separate ablation sessions were performed.

The patient was supine with the neck fully exposed. The target nodule location and its adjacent structures were evaluated under ultrasound and the puncture route was pre-designed. All procedures were performed under aseptic conditions and local anesthesia with 1% lidocaine.

When the nodule was located at the upper or lower pole of the thyroid or adjacent to important structures such as the vagus nerve, trachea, or esophagus, hydrodissection was chosen to prevent accidental injury (12), as follows. A 21-gauge needle was percutaneously inserted to the space between the thyroid capsule near the target nodule and the surrounding structures, and then 0.9% saline was injected to separate the structures around the thyroid.

During the procedure, the cystic component in the nodule was aspirated through an 18-gauge percutaneous needle. The treatments were conducted in accordance with previous reports (13–15). Power outputs of 30 and 3W were applied during MWA and LA, respectively. For MWA, after the antenna was inserted into the relatively large nodules under ultrasound guidance, the moving shot technique was used to finish the ablation. That is, each nodule was divided into multiple small conceptual units and was treated in a unit-by-unit manner by moving the antenna (4, 7). The ablation procedures were monitored by real-time ultrasound, and ablations were not terminated until the transient hyperechoic cloud caused by the gas covered all units of the nodule (16). For LA, up to 4 needles and optic fibers were inserted in a single treatment session. The number of needles and their spatial layout depended upon the size, shape and location of the nodule. A total energy of 1,600–1,800 Joules per fiber was delivered in each illumination and several consecutive illuminations could be performed with a pullback technique during the same treatment session depending on the size of the nodule.

The ablation operative durations were recorded and the energy calculated. To prevent recurrent laryngeal nerve injury, the patient was questioned for short answers between intervals of ablation. If the patient's voice changed, ablation was stopped immediately.

Post-operatively, the ablation area was covered with gauze for 6 h and the patients were observed in the hospital during this time. If there were no signs of complications and the patient's vital signs were normal, they were allowed to leave the hospital.

### Post-ablation Assessment

The success of ablation was assessed immediately afterward via contrast-enhanced ultrasonography, and included the ablative range, nodule echo, blood flow, and non-ablated portion. At postoperative 3, 6, and 12 months during the first year of follow-up, and every 6 months thereafter, the following were noted: volume of the ablated nodules, symptom scores and cosmetic scores, and laboratory tests results such as T3, FT4 and thyroid stimulating hormone.

The volume reduction rate (VRR) was the percentage change in volume after the BTN ablation. The VRR was calculated as a percentage as follows: (initial volume − final volume) × 100/initial volume.

Complications associated with the MWA and LA ablations were in accordance with the report standard of the Society of Interventional Radiology (17, 18). Major complications were considered voice changes, sympathetic nerve injury, and nodule rupture with or without infection. Minor complications included hemorrhage or hematoma, vomiting, skin burns, and thyroid function changes. Side effects included pain, coughing, and mild fever during the perioperative and follow-up periods. The patients graded themselves on the pain experienced during ablation, with a score ranging from 0 (nil) to 5 (most severe).

### Statistical Analysis

Data analyses were performed using SPSS statistical software version 22.0 (SPSS, Chicago, IL). Graph drawing was done using GraphPad Prism software version 8.0 for Windows (GraphPad Software, San Diego, California, USA).

To reduce the confounding bias that is inherent in a non-randomized retrospective experiment, this study performed propensity score matching to balance the preliminary data of the MWA and LA groups. The patient's propensity score was measured by a multivariate logistic regression model using the following baseline characteristics as covariates: gender, age, initial nodule volume, and follow-up period. Categorical variables are expressed as frequencies, and continuous variables as mean ± standard deviation. After the 1:1 match, qualitative variables were analyzed using the chi-squared test or Fisher's exact test. Quantitative variables were analyzed by Student's *t*-test. A *P* value < 0.05 was considered statistically significant.

## Results

### Patient

From June 2015 to June 2018, 318 patients conformed to the inclusion criteria for this study (Table 1). Five patients each underwent 2 separate ablation sessions, because the target BTNs were located in both lobes of the thyroid. Therefore, the total number of treated nodules was 328, specifically 160 (85 large, 75 small) treated with MWA, and 168 (90 large, 78 small) treated with LA.

**Table 1:** Baseline characteristics of patients with BTNs (overall, large, or small) treated with MWA or LA after propensity-score matching.

	**MWA**			**LA**			***P***		
	**Overall**	**Large**	**Small**	**Overall**	**Large**	**Small**	**Overall**	**Large**	**Small**
Subjects, *n*	160	85	75	160	85	75	–	–	–
Female,%	70.6	71.8	69.3	75.0	76.5	73.3	0.728	0.612	0.646
Age	42.9 ± 17.7	41.5 ± 21.1	44.2 ± 18.4	44.5 ± 21.4	43.3 ± 22.5	45.6 ± 23.0	0.467	0.591	0.681
Volume, mL	12.7 ± 5.1	16.7 ± 4.5	8.1 ± 4.8	13.1 ± 4.7	17.2 ± 4.0	8.4 ± 4.4	0.466	0.445	0.691
Symptom score	5.8 ± 2.5	6.1 ± 2.7	5.3 ± 1.8	6.0 ± 1.9	6.4 ± 2.2	5.4 ± 2.0	0.421	0.428	0.748
Cosmetic score	3.0 ± 0.8	3.2 ± 0.6	2.8 ± 0.8	2.9 ± 0.9	3.1 ± 0.7	2.7 ± 1.0	0.294	0.319	0.500
FU period, mo	14.3 ± 6.5	14.4 ± 6.5	14.1 ± 6.2	14.1 ± 6.2	13.9 ± 5.9	14.2 ± 6.0	0.778	0.600	0.920

*Values are reported as mean ± SD. MWA, microwave ablation; LA, laser ablation; FU, follow-up; mo, months*.

After the propensity score matching, the two groups both had 85 large nodules and 75 small nodules. The initial volumes of the nodules in the MWA and LA groups were 12.7 ± 5.1 and 13.1 ± 4.7 mL, respectively. After the ablations, each patient's thyroid laboratory tests results, including thyroid stimulating hormone, T3 and FT4 were within the normal range.

### Ablation Procedure

Among the total 320 nodules treated, hydrodissection was performed in 132 (41.3%) treatments: 62 (38.8%) by MWA, and 70 (43.8%) by LA. The moving shot technique was used in 129 (80.6%) treatments in MWA. The energy of ablation applied overall to BTNs in the MWA and LA groups was statistically similar (40.1 ± 26.2 KJ cf. 35.1 ± 23.3 KJ), as was the energy of ablation applied specifically to large (50.0 ± 23.6 KJ cf. 44.0 ± 21.1 KJ) and small (33.0 ± 20.8 KJ cf. 28.2 ± 14.1 KJ) nodules (Table 2). The ablative time of the MWA group overall (21.1 ± 9.1 min), and for large and small nodules specifically (26.5 ± 12.8 min and 17.9 ± 8.8 min, respectively) was significantly less than that of the LA group (47.9 ± 25.0 min, 54.7 ± 23.8 min, and 39.0 ± 26.6 min; *P* < 0.01, all; Table 2).

**Table 2:** Outcomes in the MWA and LA groups overall and large and small BTNs, specifically.

		**MWA**			**LA**			***P***		
		**Overall**	**Large**	**Small**	**Overall**	**Large**	**Small**	**Overall**	**Large**	**Small**
Subjects, *n*		160	85	75	160	85	75	–	–	–
PO 3 mo	VRR, %	54 ± 40	55 ± 37	53 ± 41	56 ± 28	57 ± 31	55 ± 27	0.605	0.703	0.725
	SS	5.5 ± 1.8	5.8 ± 1.9	5.1 ± 1.7	5.8 ± 2.0	6.2 ± 2.0	5.2 ± 1.8			
	CS	2.8 ± 1.1	3.0 ± 0.8	2.5 ± 1.2	2.8 ± 1.0	3.0 ± 1.0	2.6 ± 1.1			
PO 6 mo	VRR, %	70 ± 22	68 ± 24	72 ± 21	75 ± 26	77 ± 31	73 ± 26	0.064	0.036	0.796
	SS	4.6 ± 2.2	5.0 ± 2.2	4.4 ± 2.0	4.7 ± 2.1	5.2 ± 1.9	4.3 ± 2.3			
	CS	2.2 ± 0.9	2.4 ± 1.1	2.0 ± 0.9	2.3 ± 1.1	2.5 ± 1.2	2.0 ± 1.3			
PO 12 mo	VRR, %	75 ± 30	72 ± 25	77 ± 24	79 ± 33	81 ± 30	76 ± 33	0257	0.035	0.832
	SS	4.2 ± 1.8	4.5 ± 1.6	4.0 ± 1.8	4.1 ± 1.9	4.7 ± 1.7	3.8 ± 2.0			
	CS	1.8 ± 1.2	2.0 ± 1.3	1.7 ± 0.7	1.9 ± 1.0	2.2 ± 0.9	1.7 ± 0.8			
Last FU	VRR, %	83 ± 32	79 ± 26	85 ± 25	88 ± 25	87 ± 25	89 ± 28	0.120	0.042	0.358
	SS	3.5 ± 1.6	3.9 ± 1.9	3.2 ± 1.5	3.6 ± 1.9	4.0 ± 1.8	3.2 ± 1.8			
	CS	1.5 ± 0.8	1.6 ± 1.0	1.4 ± 0.7	1.6 ± 0.7	1.8 ± 0.8	1.4 ± 0.5			
Total energy, kJ		40.1 ± 26.2	50.0 ± 23.6	33.0 ± 20.8	35.1 ± 23.3	44.0 ± 21.1	28.2 ± 14.1	0.072	0.082	0.100
Ablation time, min		21.1 ± 9.1	26.5 ± 12.8	17.9 ± 8.8	47.9 ± 25.0	54.7 ± 23.8	39.0 ± 26.6	0.000	0.000	0.000

*Values are reported as mean ± SD*.

*MWA, microwave ablation; LA, laser ablation; FU, follow-up; mo, months; PO, postoperative; VRR, volume reduction rate; SS, symptom score; CS, cosmetic score; kJ, Kilojoules*.

### Follow-Up Evaluation

**Figure 1:**
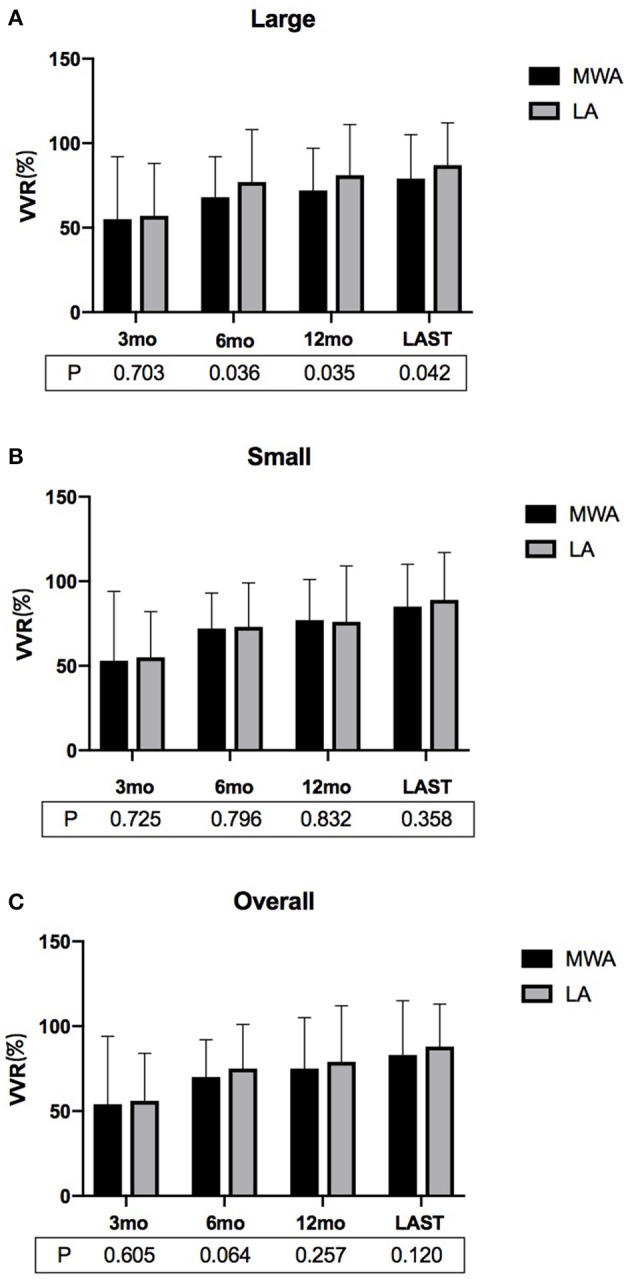
The VRRs of **(A)** large, **(B)** small and **(C)** overall BTNs after MWA or LA at the postoperative follow-ups. For large nodules, the VRR associated with LA at ≥6 months was significantly greater than that of MWA (VRR, volume reduction rate; MWA, microwave ablation; LA, laser ablation; mo, months).

**Figure 2:**
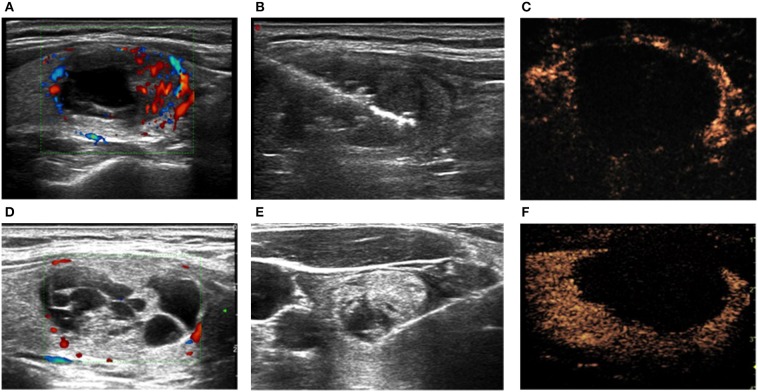
BTNs **(A,D)** before, **(B,E)** during, and **(C,F)** immediately after ablation in the **(A–C)** LA and **(D–F)** MWA groups. **(C,F)** Contrast-enhanced ultrasound.

In each of the MWA and LA groups, the volumes of the ablated nodules at each follow-up were significantly less than that at the baseline (*P* < 0.01, all). Specifically, in the MWA group, the volumes of the ablated nodules at 3, 6, and 12 months and at the last follow-up were 5.8 ± 4.0, 3.9 ± 2.3, 3.2 ± 1.8, and 2.8 ± 2.2 mL, respectively, which were much less than that before ablation (12.7 ± 5.1 mL; *P* < 0.01). In the LA group, the volumes of ablated nodules at 3, 6, and 12 months and at the last follow-up were 5.8 ± 4.6, 3.3 ± 2.8, 2.8 ± 1.9, and 2.2 ± 1.5 mL, which were much less than before ablation (13.1 ± 4.7 mL; *P* < 0.01). There was no significant difference in the overall VRRs of the MWA and LA groups at each of the follow-ups (3, 6, and 12 mo, and last follow-up). However, for large nodules, after the 6-month follow-up, the VRRs of the LA group were significantly higher than that of the MWA (Figures 1, 2; Table 2).

In both the MWA and LA groups, the symptom scores and cosmetics scores at each follow-up were less than that prior to the ablation, although the difference was not always significant (Tables 1, 2). However, the differences were statistically significant at the 6-month follow-up and after (*P* < 0.01; Table 2).

In the MWA group, the symptom score at baseline was 5.8 ± 2.5, and at postoperative 3, 6, and 12 months and last follow up the symptom scores were 5.5 ± 1.8, 4.6 ± 2.2, 4.2 ± 1.8, and 3.5 ± 1.6, respectively. In the LA group, the symptom score at baseline was 6.0 ± 1.9, and at postoperative 3, 6, and 12 months and last follow up the symptom scores were 5.8 ± 2.0, 4.7 ± 2.1, 4.1 ± 1.9, and 3.6 ± 1.9.

In the MWA group, the cosmetic score at baseline was 3.0 ± 0.8, and at postoperative 3, 6, and 12 months and last follow-up the symptom scores were 2.8 ± 1.1, 2.2 ± 0.9, 1.8 ± 1.2, and 1.5 ± 0.8, respectively. In the LA group, the symptom score at baseline was 2.9 ± 0.9, and at postoperative 3, 6, and 12 months and last follow-up the symptom scores were 2.8 ± 1.0, 2.3 ± 1.1, 1.9 ± 1.0, and 1.6 ± 0.7.

### Safety Assessment

There were five (1.6%) major complications in this study, all of which were voice changes. All patients showed hoarseness. One patient each in the MWA and LA groups recovered within 3 months after using corticosteroids and physical therapy. The rest recovered without any treatment within 3 days after the ablation. Seven (2.2%) patients had the minor complication of local formation of hematoma during the ablation, which disappeared within a week without any sequelae.

Fifty-nine patients (18.4%) experienced various degrees of pain during the ablation; 20 in the MWA group and 39 in the LA group. Of those who experienced pain in the MWA and LA groups, the pain scores were 2.2 ± 0.9 and 2.4 ± 1.3 (*P* > 0.05).

While slight pain was common in the ablation area or in the head, ears, shoulders, or teeth, especially when the ablated lesion was close to the skin, no patient needed to stop the ablation due to intolerance. The incidence of major complications and side effects in the LA group was higher than that of the MWA group, and minor complications in the MWA group exceeded that of the LA group, but the differences were not statistically significant (Table 3).

**Table 3:** Complications and side effects in the MWA and LA groups in the peri-ablation and follow-up periods.

	**MWA**			**LA**			***P***		
	**Large**	**Small**	**Total**	**Large**	**Small**	**Total**	**Large**	**Small**	**Total**
Subjects, *n*	85	75	160	85	75	160	–	–	–
Voice change	2	0	2	1	2	3	0.914	0.818	0.937
Hematoma	3	2	5	1	1	2	0.829	0.908	0.813
Pain	10	10	20	23	16	39	0.162	0.491	0.135

*MWA, microwave ablation; LA, laser ablation*.

## Discussion

Ultrasound-guided percutaneous MWA and LA are both minimally invasive and common treatments for patients with BTNs. A preliminary clinical study with a short-term follow-up showed that MWA is a safe and effective technique for the treatment of BTNs (19). Another retrospective study showed that LA is a clinically effective, repeatable, and efficient outpatient treatment that is well-tolerated and is associated with a low risk of major complications (20). However, there has been no previous systematic comparative study concerning whether one of these ablation methods is superior to the other. Thus, the present study retrospectively compared MWA and LA in terms of treatment efficacy and complications/side effects for single-session ablation of BTNs, and provides information for clinical decision-making.

The principle of MWA is that microwave energy is absorbed by charged molecules and particles in the target tissue, which then vibrate violently. The thermal effect is a quick rise in temperature (to local maximums of 100–150°C) with protein denaturation, tissue cell coagulation, dehydration, and necrosis, to achieve the therapeutic purpose. LA involves radiation of the target tissue, whereby light energy is transformed to kinetic energy with local temperatures rising to above 200°C; the local tissue coagulates, becomes necrotic, charred, or is even vaporized.

In MWA, to complete the ablation procedure the moving shot technique was usually employed after the antenna was advanced into the target nodules under ultrasound guidance. Relative to the fixed shot, the moving shot was more flexible. The size of the ablation unit could be adjusted by changing the speed at which the tip of the antenna was moved. Important adjacent structures around the nodule could be protected by speeding up the movement and using a relatively small ablation unit. In the safe area of the nodule we could extend the ablation time and adopt a larger ablation unit. For a blood-rich area it was also possible to make the tissue necrosis more thoroughly by slowing down the movement, ensuring the safety and long-term efficacy of the ablation.

In the present study, potential confounders or selection bias between the MWA and LA groups were minimized via a one-to-one propensity score matching system (21, 22). In addition, to objectively compare the efficacy and safety of the MWA and LA treatments, only one ablation session was performed on the target nodule. No significant differences were found in the mean VRRs of the two groups. However, for large nodules specifically, the LA group achieved a higher VRR than did the MWA group at the 6- and 12-month follow-ups, and at the last follow-up. This result was superimposable on a study of comparison between radiofrequency and laser ablation, in which a greater volume reduction of nodules with dimensions ≥30 mL treated with laser ablation was shown than those treated with radiofrequency ablation (23). This may be because when a large nodule is ablated, LA involves the insertion of multiple needles (up to 4), and thus multiple sources emit energy simultaneously. The nodules are more uniformly heated, and the synergistic effect results in a stable and reliable ablation range. The neck symptoms and cosmetic problems of the patients in both groups were eliminated by the sixth month of follow-up. Due to the difference in the power of ablation, the average ablation time required for MWA was shorter than for LA.

The rates of complications and side effects, especially the former, were low in both groups in this study. The MWA and LA groups were statistically comparable with regard to major or minor complications, or side effects. Voice change after ablation was the most common major complication. Five patients experienced hoarseness, and 1 patient in each of the 2 groups did not recover quickly. Such patients may have suffered recurrent laryngeal nerve injury during the ablation. These patients gradually recovered with medications and physical therapy. The voice changes in the other 3 patients may have been due to hemorrhage (24) or the transient rise in temperature, rather than neuronal necrosis caused by heat during the ablation. This often recovers naturally within days and requires no treatment.

The following cautions may help minimize voice change complications after thyroid ablation. First, a careful ultrasound examination of the target nodule and adjacent structures is required. When the nodule is located at the upper or lower pole of the thyroid gland or near important structures such as the vagus nerve, trachea, or esophagus, hydrodissection is advised to prevent accidental injury. Second, a short pause during the thermal ablation will reduce heat conduction, especially during the ablation of large BTNs. During the pause, the patient can be asked questions that require short answers, so that a voice change will be apparent. Finally, the energy should be emitted only after the tip of the ablation needle or antenna in the target nodule is clearly monitored by ultrasound (7).

Regarding minor complications, 7 patients in the present study experienced perioperative local hematoma formation in the ablation zone. The rate was slightly higher in the MWA group than the LA. This may be because the 16G antenna used in MWA is thicker than the ablation needle in LA. The 16G antenna carries a higher risk of vascular injury and bleeding in the puncture route, leading to the formation of hematoma. In addition, the temperature of LA is higher compared with MWA, and thus coagulation and hemostasis is better and hematoma is less likely. All hematomas were completely absorbed during the 1-week observation period.

The side effects in this study included various degrees of pain during the ablation. The number of patients with pain in the LA group was higher compared with the MWA group and the pain score was slightly higher. This is probably due to more heat sources in LA, and the heat spreads wider and temperature is higher than in MWA. In addition, the polytetrafluoroethylene (PTFE) coating on the surface of the MWA antenna shaft has poor thermal conductivity. Therefore, the heat generated by the tip of the antenna is transmitted less along the shaft to the patient's skin where there are many sensory nerves. This may also be why patients have relatively less pain during MWA compared with LA.

It was not an objective of the present study to compare the efficacy and safety of ablations of BTNs, whether MWA or LA, with surgical treatments, but such a study would be informative. The present study is limited by being retrospective, and the follow-up period was short. However, the preliminary evidence warrants prospective randomized controlled trials and longer follow-ups for confirmation.

## Conclusion

Percutaneous ultrasound-guided MWA and LA are both safe and effective techniques for the clinical treatment of BTNs. Either MWA or LA can significantly reduce the nodule volume, and improve patients' neck symptoms and appearance, with little risk of complications or side effects. The efficiency of MWA is higher than that of LA, but for the treatment of nodules ≥ 13 mL, the VRR of LA was greater at 6 months and at subsequent follow-ups.

## Data Availability Statement

The datasets generated for this study are available on request to the corresponding author.

## Ethics Statement

The studies involving human participants were reviewed and approved by Third Xiangya Hospital of Central South University. The ethics committee waived the requirement of written informed consent for participation.

## Author Contributions

PZ: conceptualization, writing—review and editing, and supervision. Y-FS: formal analysis, investigation, project administration, and writing—original draft. Y-FZ: resources. W-GL: software. S-MT: validation. Y-PL: visualization.

### Conflict of Interest

The authors declare that the research was conducted in the absence of any commercial or financial relationships that could be construed as a potential conflict of interest.
